# Supplementary material for: Breaking a dative bond with mechanical forces
Source: Nat Commun. 2021 Sep 24;12:5635. doi: 10.1038/s41467-021-25932-6 (PMC8463581; doi:10.1038/s41467-021-25932-6)
Supplement: Supplementary file 1 — Supplementary Information [file 41467_2021_25932_MOESM1_ESM.pdf]

Supplementary Information

Breaking a Single Chemical Bond with Mechanical Forces

Authors: Pengcheng Chen,[†] Dingxin Fan,[†] Yunlong Zhang,[*] Annabella Selloni, Emily A. Carter, Craig B. Arnold, David C. Dankworth, Steven P. Rucker, James. R. Chelikowsky,[*] and Nan Yao[*]

[†] *These authors contribute equally*
* *To whom correspondence should be addressed: E-mail: nyao@princeton.edu; yunlong.zhang@exxonmobil.com; jrc@utexas.edu*

Supplementary Methods

Force Calculation

We model the CO-FePc complex, the probe tips and the Cu(111) substrate explicitly and employ an *ab initio* real-space, pseudopotential density functional theory (DFT) code, PARSEC[1], to compute interatomic forces. The net force acting on atom a, $\vec{F}_{net,a}$, is calculated by applying the Hellmann-Feynman theorem[2,3] to the total ground state energy:

$$\vec{F}_{net,a} = -\int \rho(\vec{r}) \frac{\partial V_{loc}^{a}(\vec{r}-\vec{R}_a)}{\partial \vec{R}_a} dr - 2\sum_{lm,n} < \phi_{lm}^{a} | V_l - V_{loc} | \phi_{lm}^{a} > \frac{\partial G_{lm}^{a,n}}{\partial \vec{R}_a} - \frac{\partial E_{i-i}}{\partial \vec{R}_a} \quad (1)$$

In equation (1), the first term[4] is the local force component, where $\rho(\vec{r})$ is the valence charge density, $\vec{R}_a$ is the position of atom a, $V_{loc}^{a}(\vec{r}-\vec{R}_a)$ is the local pseudopotential component of atom a. The second term[5] is the nonlocal force component, ϕ_{lm}^{a} is the lth radial atomic pseudo-wavefunction multiplied by the lmth spherical harmonic, $V_l - V_{loc}$ is the difference between the lth component of the ionic pseudopotential and the local ionic potential, $G_{lm}^{a,n}$ is the projection coefficient with the index n running over all occupied electronic states. The third term is the ion-ion force component, where E_{i-i} is the ion-ion interaction energy.

Structural Relaxation

We use the extended limited memory Broyden-Fletcher-Goldfarb-Shanno algorithm[6] for all structural relaxations. See Supplementary Fig 1 for details of the relaxed geometries.

(1) CO-FePc complex without a Cu(111) substrate

First, we model the CO-FePc complex in the gas phase without the presence of a copper substrate. We employ a cluster boundary condition that assumes that the electron wave functions are zero

outside a spherical domain. We set the boundary sphere radius to 1100 pm. All atoms in the system are relaxed. The Fe-C bond length equals 168 pm in the final relaxed structure.

(2) CO-FePc and FePc complex on a Cu(111) substrate

Next, we model the CO-FePc complex on the copper substrate by placing the optimized CO-FePc complex on the surface. We model the substrate by a 4-layer 8x10 Cu(111) surface and place the CO-FePc (or FePc) complex at a bridge site. We employ an orthorhombic unit cell where a = 2042 pm, b = 2211 pm. We use a 2D slab boundary condition that assumes the system to be periodic along the x and y directions. We perform the calculations at the Γ point as the system is sufficiently large to obtain a reliable density from this point alone. We set the slab width to 2160 pm and fix the bottom 2 layers of the substrate during the relaxation. The Fe-C bond length increased from 168 pm to 175 pm in the final relaxed structure of CO-FePc. Furthermore, the distances between the center Fe atom and the middle of the two bridge Cu atoms decreased by ~ 30 pm upon CO removal.

(3) Probe tips with CO-FePc complex on a Cu(111) substrate

We model the interactions between different probe tips and the CO-FePc complex on the substrate by including the tips on top of the previously optimized geometries. Here, we still use a 2D slab boundary condition and set the slab width to 2670 pm. As for probe tip modeling, while some groups have modeled the probe tip as a combination of a metal cluster with an apex functionalized tip[7-9], we obtain accurate images without including the metal cluster[10-11] despite the fact that the nominal tip apex radius is significantly larger than these theoretical models. We indeed find that including the Cu cluster has a negligible effect on the interaction energy as a function of tip-sample distance for both Cu tip (tests done on Cu_2N and graphene)[12] and CO tip (tests done on benzene)[13]. In agreement with our finding, a recent study on forces acting on a CO tip obtained reliable results

by including only a single CO molecule[14]. In addition, we compute the tip-sample interaction energies for different tip conformations as a function of tip height, which is defined as the distance between the front atom of the tip and the average height of the FePc complex (excluding the decorated CO). For Cu tip, we tested Cu_2 and Cu_4 tips (Supplementary Fig 2 a); for CO tip, we test CO, CuCO, Cu_2CO and Cu_4CO (Supplementary Fig 2 b). In both cases, we do not see a significant variation in the interaction energy. Therefore, we confirm that our previous conclusion is still valid for the CO-FePc molecule.

(3.1) Cu tip

We model the Cu tip first because as there are less variables involved in terms of modelling it when compared to the CO tip. In the presence of the Cu tip, we relax the system again including a previously optimized Cu_2 cluster on top of the complex. In our optimized model structure, Fe, C, O and two Cu atoms are lined vertically along the center axis of the molecule.

(3.2) CO tip

When modeling a CO-functionalized tip, we use a previously optimized Cu-CO cluster. A direct calculation of the rupture force is difficult as the position of the tip relative to the CO-FePc complex is unknown in the three-dimensional space and more than one solution is possible for a given rupture force. To estimate the forces, we first optimize the vertically aligned tip-sample-substrate system with the tip above the center Fe atom. However, we find that varying the tip height in this vertically alighted geometry cannot rupture the dative bond. Next, we break the symmetry by displacing the tip horizontally by a small amount (~135 pm) from the equilibrium position and then adjust the tilting angles of the two COs so that the O-O distance (~250 pm) is kept constant. This allows for the existence of lateral forces. We then perform structural relaxation again. Once the new equilibrium structure is obtained, we compute the spatial distribution of the

forces by manually displacing the CO tip to different sites while keeping all atomic positions fixed. Note that, the O-O distance is no longer constant in the calculations of the force distribution. We expect the calculated forces to be overestimated as the tip moves further away from the equilibrium position owing to the fixed positions.

In order to ensure that our equilibrium structure is reliable, we perform additional structural relaxation calculations as the CO tip horizontally approaches the center of the sample molecule. For simplicity, we exclude the substrate in our calculation as our objective is to obtain a general trend of the bending motion of the two COs. We illustrate this process at different tip-sample separation distances in Supplementary Fig 3. The relaxed atomic coordinates for all the conformations are given in Supplementary Data 1-6. These structures are in good agreement with previous studies[15-16]. As shown in Ref. [16], when the COs are close to each other, they bend due to repulsion while the general orientation of the two COs remains the same (parallel) as when they bend due to attraction.

Image Simulations

We employ a frozen density embedding theory (FDET) method for image simulations. Details about FDET and previous applications can be found in Ref [10-13,17]. We also apply a tip tilting correction[18] for the CO tip (Eq. 2). We compute the displacement of the tip in x and y directions, $\vec{\Delta}_{lat}(x,y)$, by assuming a linear relationship between the lateral force, $\vec{F}_{lat}(x,y)$, and the lateral displacement:

$$\vec{\Delta}_{lat}(x,y) = \frac{\vec{F}_{lat}(x,y)}{k_{CO}} \quad (2)$$

where k_{CO} is the lateral spring constant of the CO tip. k_{CO} is an adjustable parameter which is set to be 0.80 N/m in the main text to achieve a better agreement with the experimental images. Here, Supplementary Fig 4 shows the simulated nc-AFM images for CO-FePc (the first three rows) and FePc (the last row). The left column indicates the tip height, the top row shows the corresponding lateral spring constant, k_{CO}, of the CO tip where a large value for k_{CO} corresponds to a rigid tip and a smaller value corresponds to a flexible tip. k_{CO} is set to be 0.8 N/m for the simulated images in Figure 1 (contrast adjusted, in the main text). The tip heights are 554 pm and 300 pm in Figures 1 d and e, respectively (in the main text). Before bond breaking, we only observe a bright circular spot in the middle of the molecule on the simulated image initially (554 pm). As we decrease the tip height, the FePc outline becomes slightly visible (400 pm, 430 pm). Meanwhile, the central "bright" spot becomes much larger, and a dark circular region occurs in the middle as we decrease the lateral spring constant of the CO tip. This transition is in excellent agreement with the experimental images in Figures 2 b and c in the main text (from +200 pm to +40 pm). After bond breaking, the cross-like structure with a four-fold symmetry can be clearly visualized on the simulated image in agreement with the experimental image in Figure 1 c (in the main text).

AFM Tip Scan Speed

For AFM measurement, the noise level can be adjusted by the scan speed of the tip. The longer the tip stays on one point, the lower the noise is. However, increasing the time of each point will also increase the drift, which will result in distortion of the image and inaccuracy of the data. The scan speed is about 20 ms - 100 ms for each point, so the actual scan speed during our measurement is about 0.5 nm/s.

Supplementary Figures

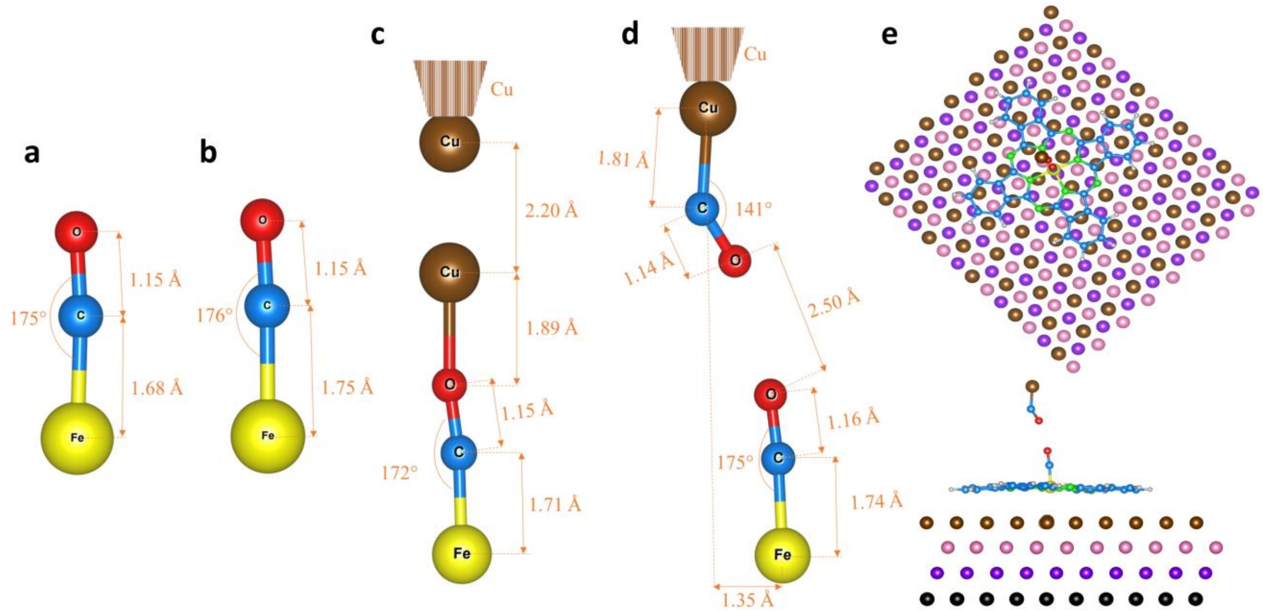

Supplementary Fig 1. Relaxed Geometries (in a-d, only Fe, decorated CO, tips and tip apexes are shown). a CO-FePc. **b** CO-FePc + Cu(111) substrate. **c** Cu apex + Cu tip + CO-FePc + Cu(111) substrate. **d** Cu apex + CO tip + CO-FePc + Cu(111) substrate. **e** top view and side view of the relaxed geometry including the substrate in **d**. Different colors are used for the Cu(111) substrate to illustrate different layers. 1st layer: brown, 2nd layer: pink, 3rd layer: purple, 4th layer: black.

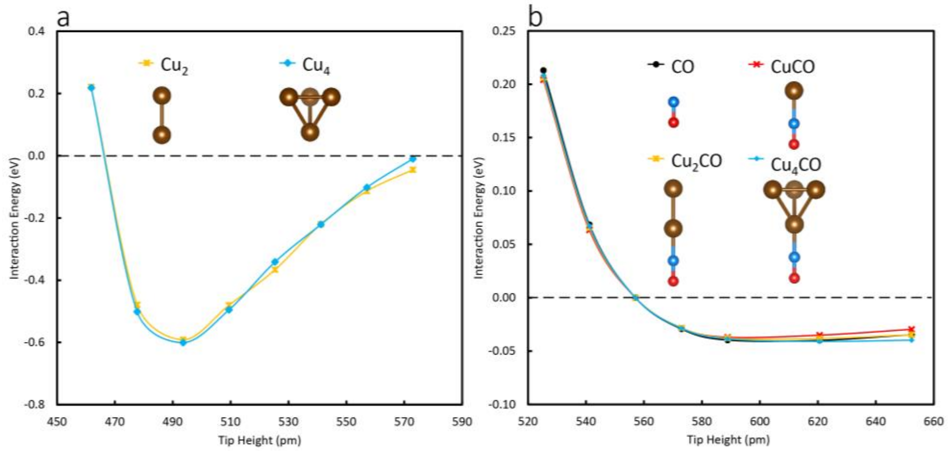

Supplementary Fig 2. Interaction energy between tip and CO-FePc. a Cu_2 and Cu_4 tips; **b** CO, CuCO, Cu_2CO and Cu_4CO tips. All the tips are placed above the decorated CO of CO-FePc. The Cu(111) substrate is not included in the calculations. Tip height is defined as the distance between the front atom of the tips and the average height of the FePc complex (exclude the decorated CO).

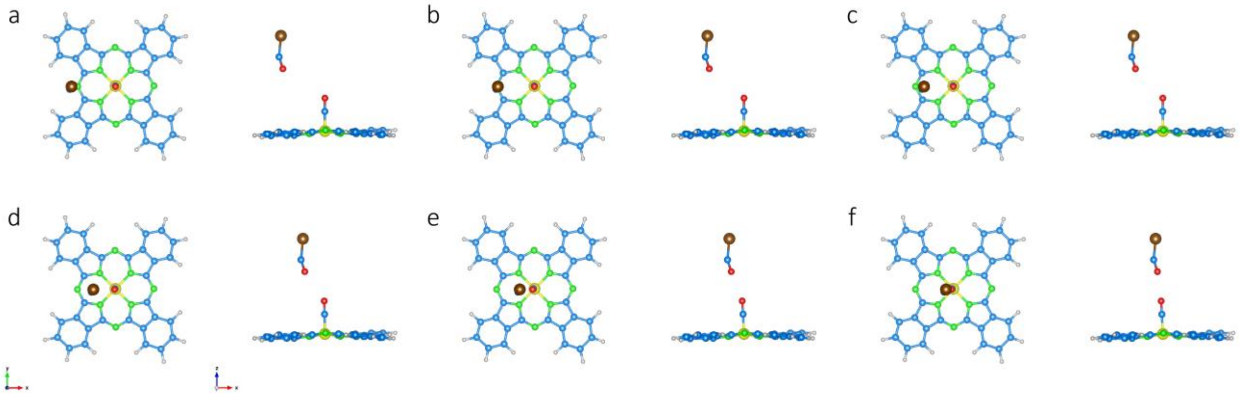

Supplementary Fig 3. Relaxed conformations of the Cu apex + CO tip + CO-FePc system at different tip-sample separation distances (left: top view; right: side view). The horizontal distances between the Cu apex and the center Fe atom are: (a) 382 pm, (b) 318 pm, (c) 255 pm, (d) 191 pm, (e) 127 pm and (f) 64 pm. The relaxed atomic coordinates are given in Supplementary Data 1-6.

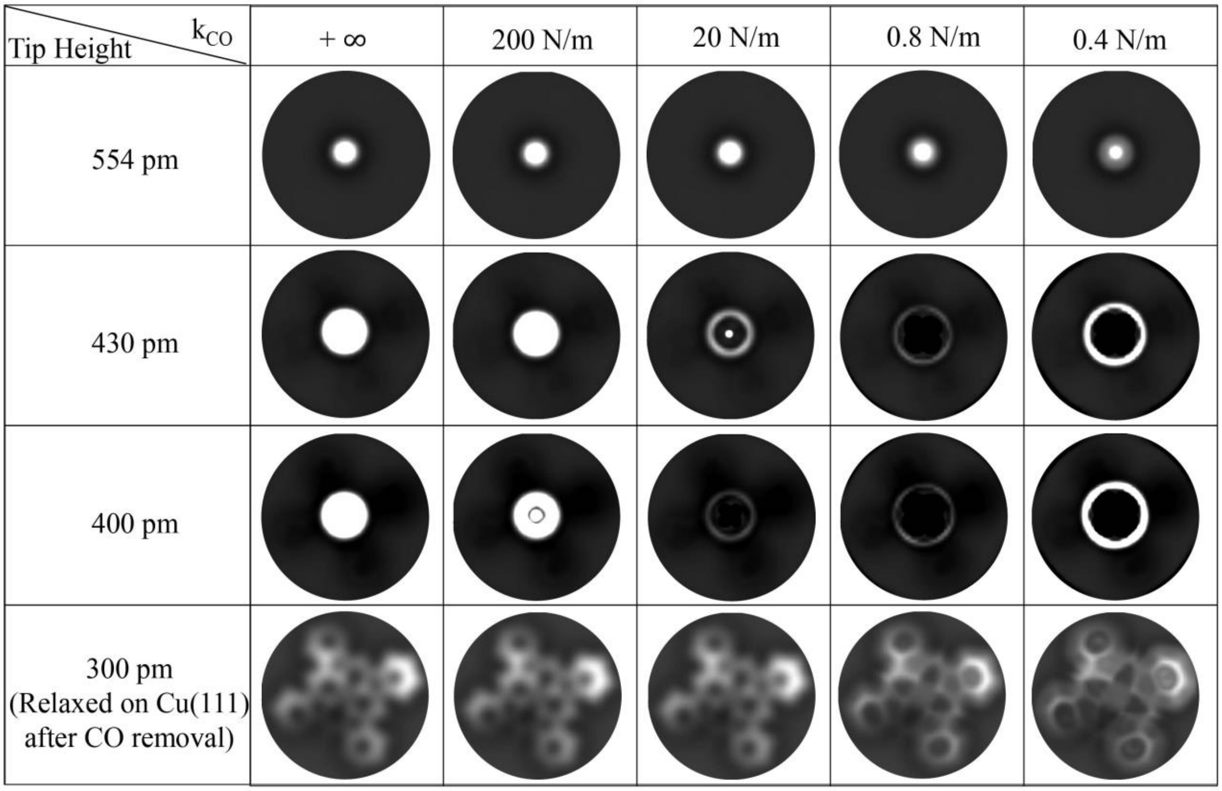

Supplementary Fig 4. Simulated nc-AFM images. The left column indicates the tip height. The top row shows the corresponding lateral spring constant of CO tip.

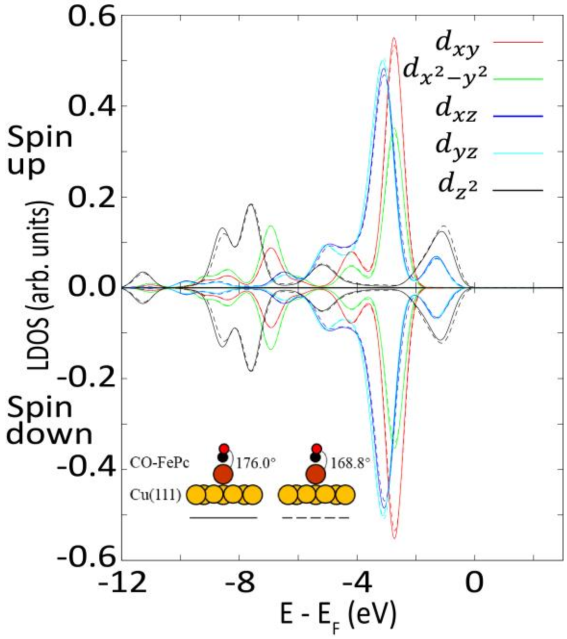

Supplementary Fig 5. **Spin-polarized electron density of states projected onto the center Fe atom of the CO-FePc on Cu(111) system.** The solid curve corresponds to the equilibrium position, the dashed curve corresponds to a O-C-Fe angle of 168.8º (the Fe-C and C=O bond lengths are fixed during rotation).

Supplementary References

1. Liou, K.-H., Yang, C. & Chelikowsky, J. R. Scalable implementation of polynomial filtering for density functional theory calculation in PARSEC. *Comput. Phys. Commun.* **254**, 107330 (2020).

2. Hellman, H. G. A. Einührung in die Quantenchemie. *Deuticke, Leipzig* (1937).

3. Feynman, R. P. Forces in molecules. *Phys. Rev.* **56(4),** 340-343 (1939).

4. Kronik, L., Vasiliev, I., Jain, M. & Chelikowsky, J. R. *Ab initio* structures and polarizabilities of sodium clusters. *J. Chem. Phys.* **115,** 4322 (2001).

5. Jing, X. *et al. Ab initio* molecular-dynamics simulations of Si clusters using the higher-order

finite-difference-pseudopotential method. *Phys. Rev. B.* **50,** 12234(R) (1994).

6. Morales, J. L. & Nocedal, J. Remark on "algorithm 778: L-BFGS-B, FORTRAN routines for large scale bound constrained optimization." *ACM Trans. Math. Softw.* **38,** 1-4 (2011).

7. Hapala, P. *et al.* Mechanism of high-resolution STM/AFM imaging with functionalized tips. *Phys. Rev. B.* **90**. 085421 (2014).

8. Gross, L. *et al.* Bond-order discrimination by atomic force microscopy. *Science*. **337**, 1326 (2012).

9. Guo, C.-S., Van Hove, M. A., Zhang, R.-Q. & Minot, C. Prospects for resolving chemical structure by atomic force microscopy: a first-principles study†. *Langmuir*. **26(21)**, 16271-16277 (2010).

10. Fan, D., Sakai, Y. & Chelikowsky, J. R. Discrimination of bond order in organic molecules using noncontact atomic force microscopy. *Nano Lett.* **19(8)**, 5562-5567 (2019).

11. Fan, D., Sakai, Y. & Chelikowsky, J. R. Chemical and steric effects in simulating noncontact atomic force microscopy images of organic molecules on a Cu (111) substrate. *Phys. Rev. Mater*. **4,** 53802 (2020).

12. Lee, A. J., Sakai, Y. & Chelikowsky, J. R. Simulating contrast inversion in atomic force microscopy imaging with real-space pseudopotentials. *Phys. Rev. B* **95**, 081401(R) (2017).

13. Sakai, Y., Lee, A. J. & Chelikowsky, J. R. First-principles atomic force microscopy image simulations with density embedding theory. *Nano Lett.* **16(5)**, 3242-3246 (2016).

14. Berwanger, J., Polesya, S., Mankovsky, S., Ebert, H. & Giessibl, F. J. Atomically resolved chemical reactivity of small Fe clusters. *Phys. Rev. Lett.* **124**, 096001 (2020).

15. Weymouth, A. J., Hofmann, T. & Giessibl, F. J. Quantifying molecular stiffness and interaction with lateral force microscopy. *Science.* **343**, 1120-1122 (2014).

16. Salmeron, M. CO meets CO, one at a time. *Science.* **343**, 1083-1084 (2014).

17. Wesołowski, T. A., Shedge, S. & Zhou, X. Frozen-density embedding strategy for multilevel simulations of electronic structure. *Chem. Rev.* **115**, 5891−5928 (2015).

18. Guo, C.-S., Van Hove, M. A., Ren, X. & Zhao, Y. High-resolution model for noncontact atomic force microscopy with a flexible molecule on the tip apex. *J. Phys. Chem. C.* **119**, 1483−1488 (2015).
